# Targeted Suppression of Chaperone-Mediated Autophagy by miR-320a Promotes α-Synuclein Aggregation

**DOI:** 10.3390/ijms150915845

**Published:** 2014-09-09

**Authors:** Guobin Li, Haiying Yang, Dezhang Zhu, Hui Huang, Guoyuan Liu, Peng Lun

**Affiliations:** Department of Neurosurgery, the Affiliated Hospital of Qingdao University, Qingdao 266003, Shandong, China; E-Mails: haiying_yangqd@163.com (H.Y.); dezhzhu888@163.com (D.Z.); huih_huang@163.com (H.H.); guoyuan626liu@163.com (G.L.); pengqdlun@163.com (P.L.)

**Keywords:** miR-320a, α-synuclein aggregation, Hsc 70, chaperone-mediated autophagy, Parkinson disease

## Abstract

Chaperone-mediated autophagy (CMA) is involved in wild-type α-synuclein degradation in Parkinson’s disease (PD), and LAMP2A and Hsc 70 have recently been indicated to be deregulated by microRNAs. To recognize the regularory role of miR-320a in CMA and the possible role in α-synuclein degradation, in the present study, we examined the targeting and regulating role of miR-320 in Hsc 70 expression. We first constructed an α-synuclein-overexpressed human neuroblastoma cell line, SH-SY5Y-Syn(+), stably over-expressing wild-type α-synuclein and sensitive to an autophagy inhibitor, which exerted no effect on the expression of LAMP2A and Hsc 70. Then we evaluated the influence on the CMA by miR-320a in the SH-SY5Y-Syn(+) cells. It was shown that miR-320a mimics transfection of specifically targeted Hsc 70 and reduced its expression at both mRNA and protein levels, however, the other key CMA molecule, LAMP2A was not regulated by miR-320a. Further, the reduced Hsc 70 attenuated the α-synuclein degradation in the SH-SY5Y-Syn(+) cells, and induced a significantly high level of α-synuclein accumulation. In conclusion, we demonstrate that miR-320a specifically targeted the 3' UTR of Hsc 70, decreased Hsc 70 expression at both protein and mRNA levels in α-synuclein-over-expressed SH-SY5Y cells, and resulted in significant α-synuclein intracellular accumulation. These results imply that miR-320a might be implicated in the α-synuclein aggravation in PD.

## 1. Introduction

Parkinson’s disease (PD) is the second most common disorder of the central nervous system in humans [1], with a prominent characteristic of dopaminergic cells degeneration within the substantia nigra pars compacta (SNpc) [2] and the cytoplasmic accumulation of proteinaceous material within aggregates called Lewy bodies (LBs) [3]. The pathological intracellular protein aggregation mainly includes, but is not limited to, α-synuclein [4,5], synphilin-1 [6], and ubiquitin [7]. A strong association of α-synuclein (α-Syn) aggregation with PD is suggested by elevated synthesis and/or reduced degradation. Increased α-synuclein gene copy number [8] promotes the α-synuclein synthesis and plays a role in PD. On the other side, impaired degradation pathways responsible for α-synuclein may also be compromised in PD [9,10]. Almost all major known degradation pathways have been implicated in the degradation of α-synuclein [11,12], particularly, autophagy and proteasomal pathways are considered to play key roles in the process [13,14,15], in which both ubiquitin-proteasome system [16,17] and the autophagy-lysosomal pathway were confirmed to degrade α-synuclein [9,18]. Moreover, the chaperone-mediated autophagy (CMA) is also involved in wild-type α-synuclein degradation in PC12 and SH-SY5Y cells [19]. Therefore, more attention needs to be paid to the molecular mechanisms of the deregulation of the α-synuclein degradation in neuronal cells to uncover the pathogenesis of PD.

Autophagy is one of main cellular homeostasis mechanisms by a “self-eating” cellular machinery to degrade and recycle long-lived proteins and organelles [20]. It has been well established that α-synuclein aggregates, as well as other neuro-pathological aggregated proteins, rely to a great extent on autophagy for clearance [21]. CMA is one of the three types of autophagic degradation pathways via lysosomes [9,22]. By selectively targeting proteins containing a KFERQ peptide motif, cytosolic Hsc 70 and its associated chaperone, LAMP2A, which is the rate-limiting lysosomal receptor on lysosomes, plays key roles in CMA [23,24,25]. Though the LAMP2A levels were reported to be very low in the central nervous system (CNS) [26,27], it has been confirmed that the inhibition of CMA by downregulating LAMP-2A leads to α-synuclein accumulation [10,19]. Thus, the deregulation of LAMP-2A and Hsc 70 is important to understand the mechanism that leads to decreased CMA in PD patients.

MicroRNAs (miRNAs) are endogenous non-coding RNAs (18–22 nt) that regulate gene expression [28] in diverse cell processes in mammals [29,30,31]. Recently, miRNAs have been shown to play important roles in brain functions [32]. Some specifically expressed or enriched miRNAs in the brain have been confirmed to associate with physiological and pathological processes in brain [33,34,35], including neurodegeneration [36,37,38,39]. Recently, many miRNAs in PD brains were reported to show significant variation among the total 224 miRNAs comparison with controls [40], suggesting that miRNA deregulation may play a role in PD pathology. Furthermore, there are miRNAs being reported to play roles in the α-synuclein aggregation in PD [40,41,42], and the CMA-associated LAMP-2A and Hsc 70 are regulated during the microRNA-mediated α-synuclein degradation via CMA.

In the present study, we first constructed an α-synuclein-overexpressed human neuroblastoma cell line, SH-SY5Y-Syn(+) to evaluate the regulation by miR-320a on the CMA and their influence on the α-synuclein degradation. It was shown that miR-320a mimics transfection specifically targeted Hsc 70 and reduced its expression in both mRNA and protein levels, and the reduced Hsc 70 promoted a significantly high level of α-synuclein accumulation. This study supports the hypothesis that elevated miRNA levels in Parkinson’s disease brains will lead to reduced CMA and result in reduced CMA-mediated α-synuclein degradation. Therefore, drugs that intervene with miRNAs, which silence CMA function in PD might be a way to modify impaired α-synuclein degradation in brains.

## 2. Results

### 2.1. Construction of SH-SY5Y Cells Over-Expressing α-Synuclein

**Figure 1:**
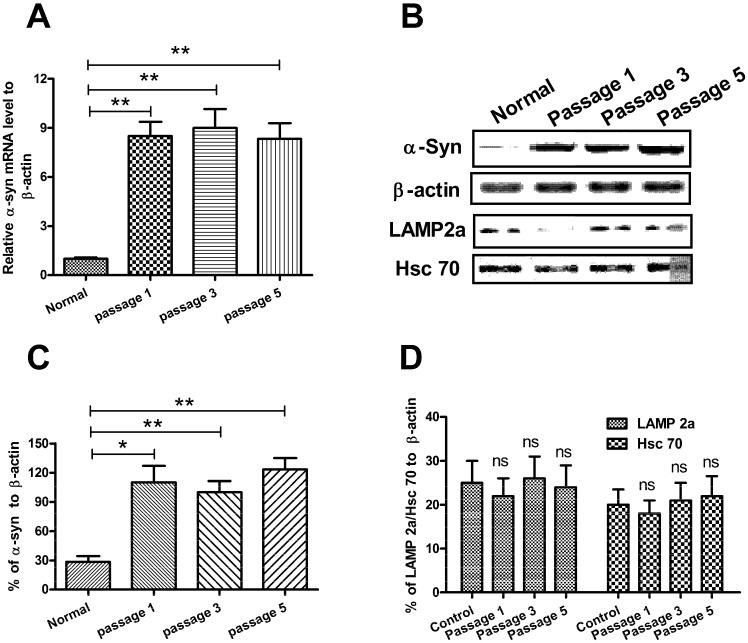
Over-expression of α-Syn in SH-SY5Ycell line. (**A**) Relative mRNA level of α-Syn in the SH-SY5Y-Syn(+) cells post various passages, compared to β-actin, revealedby quantitative real-time RT-PCR; (**B**) Western blot analysis of α-Syn, LAMP2A and Hsc 70 in the SH-SY5Y-Syn(+) cells, post various passages;(**C**) Significantly high level of α-Syn in variously propagated SH-SY5Y-Syn(+) cells; (**D**) LAMP2A and Hsc 70 protein level in the SH-SY5Y-Syn(+) cells, post various passages. Data represent the mean ±SEM of three independent experiments. Statistical significance was considered with a *p*-value <0.05 or less, * *p* < 0.05, ** *p* < 0.01, ns, no significance.

To explore the influence of CMA-targeted microRNAs on the α-synuclein degradation, we firstly established an SH-SY5Y cell line over-expressing wild-type α-synuclein, SH-SY5Y-Syn(+). Wild-type α-Syn coding sequence was amplified and cloned into a pcDNA3.1(+) vector. Stable α-Syn-over-expressed SH-SY5Y-Syn(+) cells were selected and maintained in complete medium containing 300 μg/mL G418, post the α-Syn-pcDNA3.1(+) transfection. A significantly higher and stable level of α-Syn mRNA was observed in the SH-SY5Y-Syn(+) cells post various passages than normal SH-SY5Y cells (*t*-test, *p* < 0.01 respectively) (Figure 1A). The α-Syn expression in protein level was also upregulated in the SH-SY5Y-Syn(+) cells (*p* < 0.05 for *t*-test, or *p* < 0.01 for ANOVO test, respectively) (Figure 1B,C), revealed by western blot analysis. On the other side, to confirm whether there was an influence on the expression of CMA-associated molecules, we also examined the expression of Hsc 70 and LAMP2A in protein levels by western blot assay; it was shown in Figure 1B,D that there was no significant difference in the expression of the two molecules between SH-SY5Y-Syn(+) cells and normal SH-SY5Y cells.

### 2.2. SH-SY5Y-Syn(+) Cells Are Sensitive to Autophagy Inhibitors

**Figure 2:**
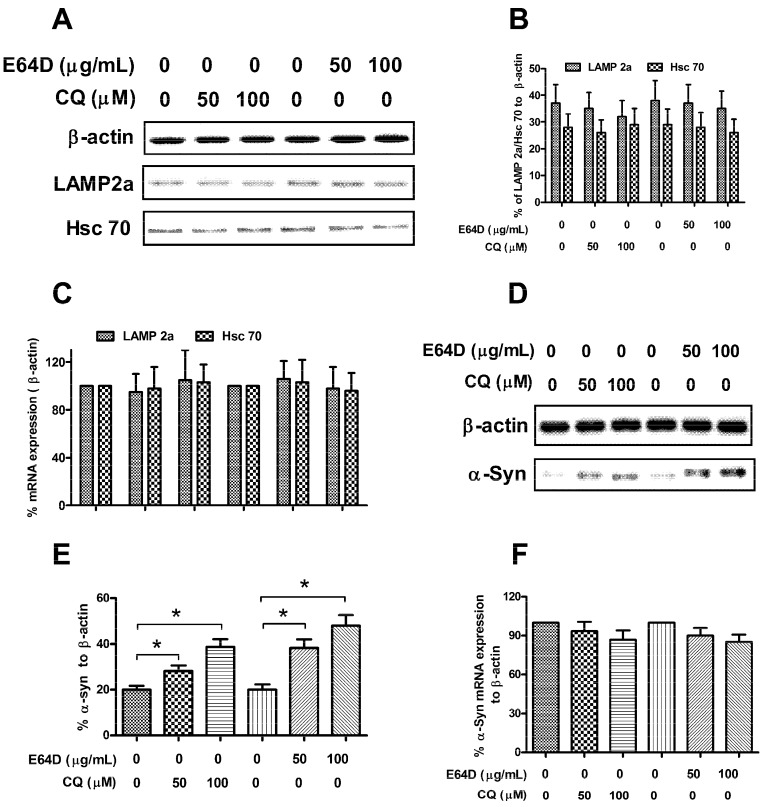
SH-SY5Y-Syn(+) cells are sensitive to the E64D or CQ. (**A**) Western blot analysis of LAMP2A and Hsc 70 expression in SH-SY5Y-Syn(+) cells post E64D or CQ treatment; (**B**,**C**) E64D or CQ treatment had no influence on the LAMP2A or Hsc 70 expression in protein level (**B**) and in mRNA level (**C**) in SH-SY5Y-Syn(+) cells; (**D**,**E**) Western blot analysis of the α-Syn accumulation in the SH-SY5Y-Syn(+) cells post E64D or CQ treatment; (**F**) α-Syn expression in mRNA level in SH-SY5Y-Syn(+) cells post E64D or CQ treatment. Data represent the mean ±SEM of three independent experiments. Statistical significance was considered with a *p*-value<0.05, * *p* < 0.05.

To investigate whether the SH-SY5Y-Syn(+) cell line is qualified to evaluate the α-Syn degradation by autophagy, we exposed the SH-SY5Y-Syn(+) cells to optimal concentrations of chloroquine (50 or 100 μM) or E64D (50 or 100 μg/mL) for 48 h, both of which inhibits the autophagic protein degeneration, by blocking lysosome fusion (chloroquine) [43] or inhibiting lysosomal protease (E64D) [44]. Neither drug significantly affected LAMP2A or Hsc 70 expression in protein level (Figure 2A,B) or in mRNA levels (Figure 2C) in the SH-SY5Y-Syn(+) cells. The α-Synuclein protein levels in the SH-SY5Y-Syn(+) cells significantly increased post the chloroquine or E64D treatment, by western blot analysis (*p* < 0.05 for *t*-test, or *p* < 0.01 for ANOVO test respectively) (Figure 2D,E). To determine whether the α-Syn increase in transcription was induced, we also determined the α-Syn expression at the mRNA level by reverse transcription reactions and quantitative polymerase chain reactions (RT-qPCR) method. And Figure 2F demonstrated that the α-Syn upregulation in protein level was not associated with increased α-synuclein mRNA levels post chloroquine or E64D treatment. Therefore, SH-SY5Y-Syn(+) cells were sensitive to chemical inhibitors on autophagic degeneration, which had no regulation on the endogenous CMA-associated molecules, LAMP2A and Hsc 70.

### 2.3. miRNA-320a Targets and Reduces Hsc 70 Expression in SH-SY5Y-Syn(+) Cells

**Figure 3:**
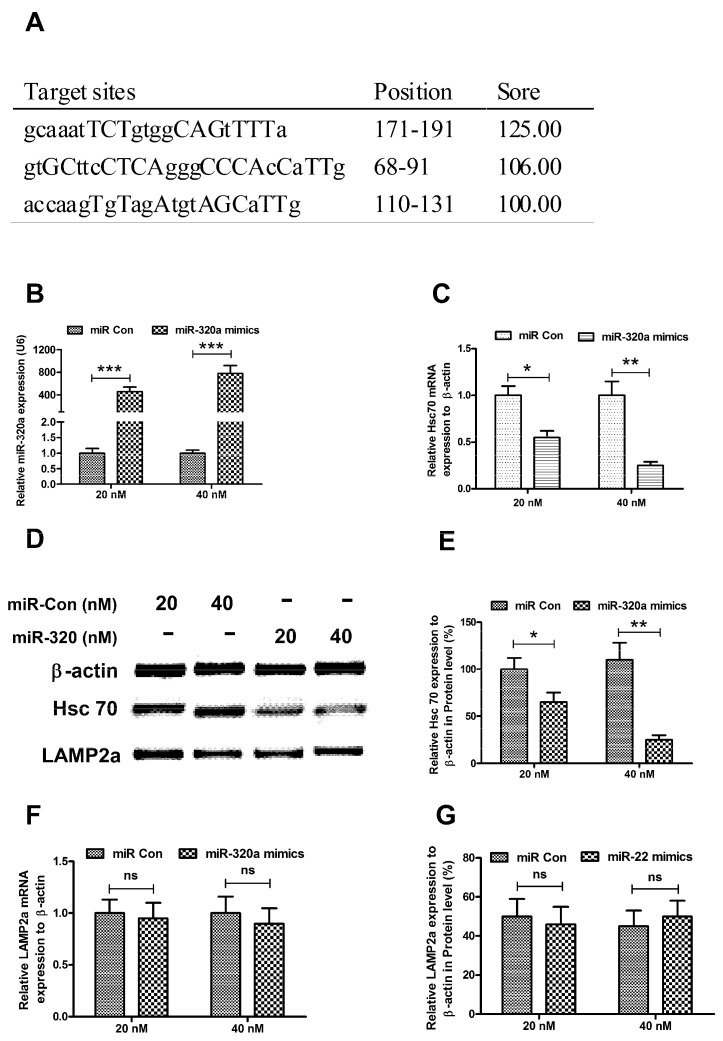
MiRNA-320a downregulates Hsc 70 expression in SH-SY5Y-Syn(+) cells. (**A**) Potential targeting sites in 3' UTR of Hsc70, predicted by miRTarBase; (**B)** Significant promotion of miR-320a level by miR-320a mimics transfection in the SH-SY5Y-Syn(+) cells; (**C**) Down-regulation of Hsc 70 in mRNA level by miR-320a mimics transfection in the SH-SY5Y-Syn(+) cells; (**D**) Western blot analysis of Hsc70 and LAMP2A in SH-SY5Y-Syn(+) cells post miR-320a mimics or miRNA control transfection; (**E**) Significant downregulation of Hsc 70 in protein level by miR-320a mimics transfection; (**F**) LAMP2A expression in mRNA level in SH-SY5Y-Syn(+) cells post miR-320a mimics or miRNA control transfection; and (**G**) LAMP2A expression in protein level in SH-SY5Y-Syn(+) cells post miR-320a mimics or miRNA control transfection. The experiments were performed separately in triplicate. Statistical significance was shown as * *p* <0.05, ** *p* <0.01, *** *p* <0.01, ns, no significance.

It has been implied that miRNA-320a is highly paired to the 3' UTR or Hsc 70 in neuronal cells [42]. In order to confirm the deregulation of miRNA-320a on Hsc 70 expression and its influence on α-Syn degradation, we evaluated the expression of Hsc 70, LAMP2A and α-Syn accumulation in the SH-SY5Y-Syn(+) cells. We found that the miRNA-320a was highly paired to three sites at the 3' UTR or Hsc 70 by miRTarBase analysis (Figure 3A). Then we elevated the miRNA-320a level in the SH-SY5Y-Syn(+) cells by transfection of miRNA-320a mimics. Figure 3B indicated a significantly high level of miR-320a in the cells post 20 or 40 nM miRNA-320a mimics transfection, compared to the miRNA control group (either *p* < 0.001). Moreover, the miRNA-320a mimics transfection could significantly downregulate the α-Syn level in mRNA level (*p* < 0.05 for 20 nM and *p* < 0.01 for 40 nM) (Figure 3C) and in protein level (*p* < 0.05 for 20 nM and *p* < 0.01 for 40 nM) (Figure 3D,E). However, the expression of the other important CMA molecule, LAMP2A was not influenced by the miRNA-320a mimics transfection (Figure 3D,F,G).

### 2.4. miR-320a Mimics Inhibited α-Synuclein Degradation in SH-SY5Y-Syn(+) Cells

**Figure 4:**
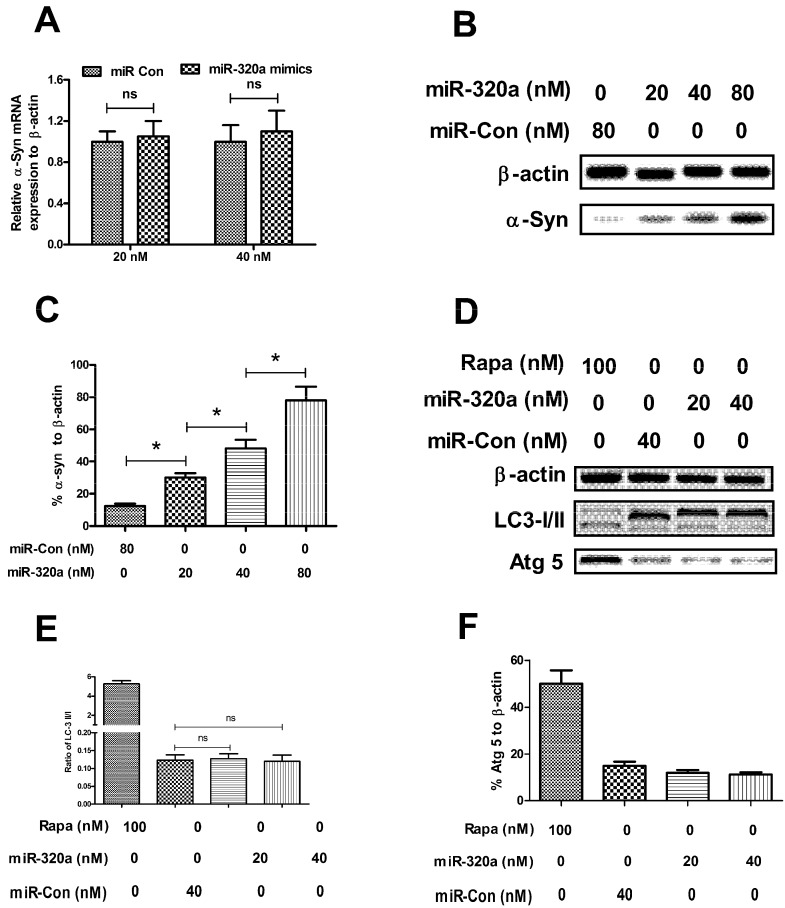
miRNA-320a inhibited α-synuclein degradation in SH-SY5Y-Syn(+) cells. (**A**) α-Syn expression in mRNA level in SH-SY5Y-Syn(+) cells post miR-320a mimics or miRNA control transfection; (**B**) Western blot analysis of α-Syn in SH-SY5Y-Syn(+) cells post miR-320a mimics or miRNA control transfection; (**C**) Significant increase in α-Syn aggragation in SH-SY5Y-Syn(+) cells by miR-320a mimics transfection; (**D**) Western blot analysis of autophagy-associated molecules, LC-3 I/II and Atg 5 in SH-SY5Y-Syn(+) cells post miR-320a mimics or miRNA control transfection; (**E**) miR-320a mimics transfection had no influence on the conversion of LC3-II to LC3-I; and (**F**) miR-320a mimics transfection had no influence on the Atg 5 expression in protein level. The experiments were performed separately in triplicate. Statistical significance was shown as * *p* <0.05, ns, no significance.

To further explore the influence of Hsc 70 knockdown by miR-320a on the α-synuclein aggregation, we determined the influence of miR-320a mimics or miRNA control transfection on the α-synuclein aggregation. It was indicated that neither 20, nor 40 nM miR-320a mimics transfection significantly regulated the α-synuclein expression in mRNA level (Figure 4A), compared to the miRNA control. However, the western blot assay revealed that the miR-320a mimics significantly induced a high level of α-synuclein aggregation in the SH-SY5Y-Syn(+) cells, with a dose-dependence and time-dependence, there was significant difference in groups with different miR-320a mimics concentration (*p* < 0.05 for *t*-test, *p* < 0.01 for ANOVA test, respectively) (Figure 4B,C). To confirm that the α-synuclein aggregation was only caused by CMA blockage, we further determined the autophagy-associated molecules, LC3-I/II and Atg 5. It was shown in Figure 4D,E that the conversion of LC3-I to LC3-II, and the expression of Atg 5 was not influenced by the miR-320a transfection, compared to the miRNA control (*p* > 0.05, respectively). Thus, we have identified the promotion of miR-320a to the α-synuclein aggregation in the α-synuclein overexpressing SH-SY5Y cells, by inhibiting the Hsc 70-mediated CMA.

## 3. Discussion

Sporadic PD is pathologically characterized with α-synuclein aggregation and formation of LBs. However, the biochemical abnormality responsible for the disease remains to be defined. Compared to familial PD, which is believed to be caused by the multiplication of the α-synuclein locus and increased α-synuclein synthesis [8], sporadic PD is normal in α-synuclein synthesis, whereas it is deficient in α-synuclein degradation, which leads to α-synuclein accumulation and aggregation [45,46]. And there is a consensus that α-synuclein aggregation is predominantly caused by impaired CMA [10,19]. Up to now, there are many reports focusing on the impaired α-synuclein degradation by CMA. On one side, various α-synuclein mutations and modifications including oxidative damage and S129 phosphorylation [47,48] lead to the blockage of α-synuclein degradation. On the other side, the deregulated CMA-associated LAMP2A and Hsc 70 have been confirmed in PD brains [10]. Further, α-synuclein aggregation is reported to be a consequence of impaired degradation by CMA [42].

Recent studies have reported, the influence of miRNAs on the various neurodegenerative disorders including Alzheimer’s disease [37] and Parkinson disease [49] via post-transcriptional regulation of protein levels. In PD, significant changes in the miRNA expression profile have been reported with numerous miRNA precursors elevated in the midbrain [40]. And some of these miRNAs, such as miR-26b, miR-106a and miR-301b (targeting Hsc 70), or miR-21, miR-224 and miR-373 (targeting LAMP2A) have been confirmed to target the 3' UTR of either Hsc 70 or LAMP2A [42]. And miR-320a was also indicated to target the 3' UTR of LAMP2A. However, miR-320a had no influence on the expression of endogenous LAMP-2A in normal SHSY5Y cells, and the luciferase reporter assay reconfirmed that miR-320a had no regulation on the 3' UTR of LAMP-2A [42].

In the present study, we firstly constructed an α-synuclein-over-expressed human neuroblastoma cell line, SH-SY5Y-Syn(+), which stably over-expressed α-synuclein, whereas the expression of CMA-associated Hsc 70 and LAMP2A had not been influenced by the over-expressed α-synuclein. Moreover, the SH-SY5Y-Syn(+) cells were sensitive to autophagy inhibitor, E64D and chloroquine; treatment with E64D or chloroquine significantly induced α-synuclein accumulation in the SH-SY5Y-Syn(+) cells, whereas the CMA-associated Hsc 70 and LAMP2A were not regulated by the over-expressed α-synuclein or the both agents. Thus, the α-synuclein-over-expressed human neuroblastoma cell line was qualified to evaluate the regulation by miRNAs on the CMA and their influence on the α-synuclein degradation. Then we screened miRNAs which targeted Hsc 70 or LAMP2A with miRTAR, and it was shown that miR-320a targeted the 3' UTR of Hsc 70. The significantly elevated miR-320a level by miR-320a mimics transfection specifically reduced the expression of Hsc 70 at both the mRNA and protein levels. Further, the reduced level of Hsc 70 by miR-320a mimics transfection attenuated the α-synuclein degradation in the SH-SY5Y-Syn(+) cells. The present study demonstrates for the first time that miR-320a targeted the 3' UTR of Hsc 70, and attenuated the role of Hsc 70 in the α-synuclein degradation. It implies that miR-320a might be involved in α-synuclein aggravation in PD.

## 4. Materials and Methods

### 4.1. Reagents, Cell Culture and Treatment

SH-SY5Y cells (human neuroblastoma cell line) purchased from ATCC were cultured in Dulbecco’s modified Eagle’s medium (DMEM) (Invitrogen, Carlsbad, CA, USA) containing 10% FBS (Invitrogen, Carlsbad, CA, USA) or maintained in DMEM supplemented with 2% FBS. E64D and chloroquine were purchased from Sigma–Aldrich (St. Louis, MO, USA) and were resolved in DMEM medium supplemented with 2% FBS. To generate an α-synuclein overexpressed cell line, the wild α-synuclein coding sequence was amplified and cloned into the multiple cloning sites between BamH I and Hind III of pcDNA3.1(+) vector. And SH-SY5Y cells were transfected with the α-Syn-pcDNA3.1(+) or control pcDNA3.1(+) vectors, and were selected in the presence of 0.5 mg/mL G418. miR-320a mimics or miRNA control (GenePharma, Shanghai, China) with a concentration of 20 or 40 nM was transfected with lipofectamine 2000 (Invitrogen, Carlsbad, CA, USA) into the SH-SY5Y cells to promote the miR-320a level.

### 4.2. RNA Isolation, Reverse Transcription, Reverse Transcription Reactions and Quantitative Polymerase Chain Reactions (RT-qPCR)

Total cellular RNA was isolated with PureLink^®^ RNA Mini Kit (Invitrogen, Carlsbad, CA, USA), and miRNAs was isolated using mirVana™ miRNA Isolation Kit (Ambion, Austin, TX, USA) according to manuals. The expression of α-Syn, Hsc 70, LAMP2A or β-actin in mRNA level was quantified by the real-time RT-PCR method with Takara One Step RT-PCT kit (Takara, Tokyo, Japan). The primers for α-Syn, Hsc 70, LAMP2A or β-actin were as follows: PF-Hsc70: 5'-TGCTGCTGCTATTGCTTACG-3', PR-Hsc70: 5'-TCAATAGTGAGGATTGACACATCA-3'; PF-Lamp2a: 5'-GTCTCAAGCGCCATCATACT-3', PR-Lamp2a: 5'-TCCAAGGAGTCTGTCTTAAGTAGC-3'; PF-β-actin: 5'-AAGGACTCCTATAGTGGGTGACGA-3', PR-β-actin: 5'-ATCTTCTCCATGTCGTCCCAGTTG-3'; PF-α-Syn: 5'-AGGACTTTCAAAGGCCAAGG-3', PR-α-Syn: 5'-TCCTCCAACATTTGTCACTTGC-3'. miRNAs were isolated using mirVana miRNA Isolation Kit (Ambion, Austin, TX, USA). RT-qPCR were performed using the MicroRNA TaqMan Reverse Transcription Kit and the TaqMan MicroRNA Assays (Applied Biosystems, Foster City, CA, USA) for mir-320a. mRNA or miRNA samples were amplified using primer sets specific for the genes of interest on a Lightcycler 480 II (Roche Diagnostics, GmbH, Germany). Relative quantification was determined using the ∆∆*C*_t_ method using *β-actin* or *U6* as reference gene [50].

### 4.3. Western Blot Analysis

Cell samples were lysed with cell lysis reagent (Promega, Madison, WI, USA) and quantified using BCA Protein Assay Reagent Kit (Pierce, Rockford, IL, USA), protein samples were separated by a 12% gradient SDS-PAGE gel, transferred to polyvinylidene fluoride membrane. Then the blotted membrane was blocked with 5% milk and respectively incubated with the rabbit polyclonal antibody to Hsc 70 (Abcam, Cambridge, UK), LAMP2A (Abcam, Cambridge, UK), α-synuclein (Santa Cruz Biotechnology, Santa Cruz, CA, USA), LC3-I/II (Sigma–Aldrich, St. Louis, MO, USA), Atg 5 (Sigma–Aldrich, St. Louis, MO, USA) or β-actin (Santa Cruz Biotechnology, Santa Cruz, CA, USA), followed by incubation with horseradish peroxidase-coupled secondary antibody (Cell Signaling Technology, Inc., Danvers, MA, USA). The proteins were detected using enhanced chemiluminescence (Thermo Scientific, Rockford, IL, USA). All immunoblots are representative of at least three independent experiments.

### 4.4. Statistical Evaluation

All data are presented as mean ± SE. For the expression analysis of Hsc 70, LAMP2A, α-synuclein, LC3-I/II, Atg 5 in mRNA or protein levels, or miR-320a between two groups, statistical evaluations are presented as mean ± SE. Data were analyzed using the Student’s *t*-test, and the statistical difference among more than two groups, data were analyzed using multivariate ANOVA test. A statistical significance was considered when *p* < 0.05. Statistical analyses were performed using SPSS 16.0 software (IBM SPSS, Inc., Chicago, IL, USA) according to the software manual.

## 5. Conclusions

In conclusion, we demonstrated that miR-320a specifically targeted the 3' UTR of Hsc 70, decreased Hsc 70 expression at both the protein and mRNA levels in an α-synuclein-over-expressed SH-SY5Y cells, and resulted in significant α-synuclein intracellular accumulation. The present study for the first time indicates that miR-320a targeted the 3' UTR of Hsc 70, and attenuated the role of Hsc 70 in the α-synuclein degradation. It implies that the miR-320a might be involved in α-synuclein aggravation in PD.
